# The efficacy of a hunger-induced two-week tube-weaning program: a retrospective study

**DOI:** 10.3325/cmj.2025.66.282

**Published:** 2025-08

**Authors:** Maria Elena Papagni, Sara Sila, Tena Niseteo, Tihana Koren, Ivana Bukovina, Mara Vukadin Petković, Zrinjka Mišak, Ana Močić Pavić, Sanja Kolaček, Iva Hojsak

**Affiliations:** 1 Section of Pediatrics, Interdisciplinary Department of Medicine, Giovanni XXIII Children's Hospital, University of Bari Aldo Moro, Bari, Italy; 2 Department of Pediatrics, Referral Centre for Pediatric Gastroenterology and Nutrition, Children's Hospital Zagreb, Zagreb, Croatia; 3 Department of Pediatrics, University of Zagreb School of Medicine, Zagreb, Croatia; 4 Department of Pediatrics, University J.J. Strossmayer School of Medicine, Osijek, Croatia

## Abstract

**Aim:**

To assess the efficacy of a multidisciplinary tube-weaning program.

**Methods:**

This retrospective cohort study enrolled children with feeding tube dependency who had not responded to standard tube-weaning interventions. All participants underwent a structured two-week multidisciplinary tube weaning delivered in a day-hospital setting at the Children’s Hospital Zagreb in the period from August 2016 to February 2023. The program was based on a hunger-induction approach and combined with mealtime therapy, sensory integration therapy, parental group therapy and individual support, nutritional counseling, and a workshop for parents. The outcomes were evaluated at the beginning of the program, the end of the program, 3-6 months after the program, and at the last follow-up.

**Results:**

The study enrolled 30 patients (mean age 2.6 ± 1.9 years; 60.0% girls). All patients had complex medical conditions and were tube-dependent for a mean of 29 ± 22 months. The majority (70.0%) had a psychomotor delay. At the end of the program, 27 (90.0%) patients showed improved oral intake, and 10 (33.3%) were completely weaned off tube feeding. During follow-up (mean 40.5 months, n = 24 children), 18 children (78.0%) were weaned off tube feeding.

**Conclusions:**

A two-week program effectively improved oral intake and resulted in a high success rate for tube weaning in the long term.

Pediatric feeding disorder (PFD) is defined as “impaired oral intake that is not age-appropriate and is associated with medical, nutritional, feeding skill, and/or psychosocial dysfunction” (1). Around 3%-10% of children from the general population experience feeding disorders (2), while up to 80%-90% of children with developmental delay may experience PFD (3).

Children or infants who are unable to adequately feed themselves and cannot receive their nutritional requirements orally need nutritional support by enteral nutrition (EN) (4). EN can be temporary or long-lasting. Nasogastric (NG) or, in rare cases, postpyloric tubes are usually chosen for temporary EN, while for longer periods, percutaneous endoscopic gastrostomy (PEG) is preferred (5).

Although EN is often essential for the survival and proper development of children with PFD, long-term EN may lead to limited oral feeding practice, significant oral-motor delays, negative food experiences, oral food refusal, altered hunger and satiation perception, and caregivers’ feeding-related anxiety (6). These factors and pre-existing medical conditions make it challenging to transition from exclusive or predominant EN to normal oral feeding. Feeding tube dependency has considerable psychosocial consequences for both the child and the caregivers, along with important financial implications due to the high costs of tube feeding devices and enteral formulas (6,7).

Tube-weaning should begin as soon as the child is medically stable and capable of starting oral feeding. Treatment strategies include different weaning models, frequently involving multidisciplinary interventions aimed at increasing oral food intake and reducing tube dependency (8). The multidisciplinary team (MDT) may include psychologists, nurses, clinical dietitians, gastroenterologists, speech and language therapists, occupational therapists, and other experts (8).

Historically, tube weaning was heavily influenced by centers’ experiences and was a slow gradual procedure that required a long time, even more than a month (9-11). Only recently, short and intense tube-weaning treatments have been described (12,13). However, there is still no consensus on the best approach. Therefore, this study aimed to summarize our seven-year experience with a short, two-week multidisciplinary intense tube-weaning program.

## PATIENTS AND METHODS

This retrospective cohort study enrolled patients who participated in a two-week tube-weaning program from August 2016 to February 2023, delivered in a day hospital setting at the Children’s Hospital Zagreb, Zagreb, Croatia.

The indications for tube feeding varied, with the largest group being children born prematurely with complications of prematurity. Other common diagnoses included genetic syndromes (eg, Pierre Robin syndrome, Down syndrome), complex congenital heart defects, and chronic pulmonary disease (Table 1). All met the criteria for PFD.

**Table 1:** Demographic characteristics at the beginning of the program (N = 30)*

Characteristic	n (%)
Age (years) at beginning of the program, mean (SD)	2.6 (1.9)
Women	18 (60.0)
The most frequent primary diagnosis	
prematurity with complications:	11 (36.0)
Pierre Robin syndrome	2 (6.6)
Down syndrome	2 (6.6)
complex heart defects	3 (10.0)
primary immunodeficiency	2 (6.6)
genetic disorders	3 (10.0)
pulmonary disease	4 (13.3)
other	3 (10.0)
Psychomotor delay	21 (70.0)
Mode of enteral nutrition delivery	
nasogastric tube	
gastrostomy	18 (60.0) 12 (40.0)
Duration of EN prior to the program, months, mean (SD)	29.0 (22.0)

*Abbreviations: SD – standard deviation; EN – enteral nutrition.

Prior to inclusion in the intensive weaning program, all had received multidisciplinary care and standard tube-weaning interventions, including behavioral strategies such as family meal participation, promotion of positive feeding experiences, and avoidance of pressure to eat. Due to persistent dependence on tube feeding, they were enrolled in the intensive multidisciplinary program.

To be eligible for the program, a child had to fulfill the following criteria: dependence on orogastric (OGT)/nasogastric tube (NGT)/PEG feeding, satisfactory medical condition, safe oral feeding as evaluated by the physician, parents’ willingness to participate in the tube-weaning program and feed their child orally, psychomotor readiness for oral feeding as assessed by a psychologist and an occupational therapist, and satisfactory nutritional status as evaluated by a clinical dietitian.

Before initiating the weaning program, patients were assessed by a pediatric gastroenterologist, clinical dietitian, psychologist, and occupational therapist. All MDT members had to unanimously decide that the child was ready to be included in the program.

We collected demographic and medical data on age, sex, gestational age, medical diagnosis, the presence of a psychomotor delay, and duration of tube feeding. Data were collected at the following time points: the first visit to the MDT or an MDT member, beginning of the program, end of the program, 3 to 6 months after the end of the program, and the last visit to the MDT. We collected data on the type of nutrition (enteral formula), the presence of oral intake, the mode of feeding (NGT or PEG), body weight (BW), BW Z-score, body height (BH), BH Z-score, body mass index (BMI), BMI Z-score, and the proportion of calories provided enterally.

The primary outcome was the proportion of children who improved their oral intake (increased their baseline oral intake/reduced their enteral intake) and who were completely weaned from EN after the two-week program.

The secondary outcomes were changes in oral intake, changes in anthropometric measures (BW, BW z-score, BH, BH z-score, BMI, and BMI z-score), and the proportion of calories given enterally at 3-6 months after the end of the program and at follow-up.

The study compared the weaned-off EN group to the not-weaned-off EN group at the end of the program in terms of baseline data, including age, sex, psychomotor delay, BW, BH, oral intake at the start of the program, and the percentage of calories given enterally.

The study was approved by the Ethics Committee of the Children’s Hospital Zagreb, Zagreb, Croatia and was performed in accordance with the 1964 Helsinki declaration and its later amendments or comparable ethical standards.

### Tube-weaning program

The tube-weaning program was developed according to the “Graz model” (6). This two-week program consists of five hours of daily activities for five out of seven days each week, for a total of 10 days. The program is strictly supervised by an MDT consisting of a pediatric gastroenterologist, clinical dietitian, psychologist, and occupational therapist. It is carried out at the day hospital in groups of 5 patients and is fully covered by health insurance.

During the program, the children are exposed to a variety of foods in a safe, pleasant, and stimulating environment supervised by the team. The program consists of medical monitoring, group (two meals per day) and individual feeding therapy, sensory integration therapy, nutritional monitoring and counselling, lectures and workshops, psychological counseling, individual and group support.

A week before the start of the program, children's tube feeding is gradually reduced by up to 50% of their current enteral intake to stimulate hunger. During the program, EN is further decreased. When and if the child shows readiness, the NG tube is removed. An individualized treatment plan is developed, taking into account each child’s medical condition, psychomotor development, nutritional status, and socioeconomic conditions. During the two-week program, parents can contact the multidisciplinary team via email at all times. After the end of two-week treatment, follow-up is continued every week for 2-4 weeks, upon which, depending on the decision of the team, further follow-up is individualized based on the child’s needs.

### Statistical analysis

The differences between categorical variables were assessed with a χ^2^ test. The normality of data distribution was tested with a Shapiro-Wilk test. Data are expressed as means and standard deviations (SD) or medians (ranges). The differences between the groups in non-categorical variables were assessed with a *t* test for independent samples or Mann-Whitney U test and, for paired samples, with ANOVA or Friedman test. Binary logistic regression was used to determine the factors associated with the success (increase in peroral intake) or weaning from EN at the end of the program and at the end of follow-up. The level of statistical significance was set at *P* < 0.05. Statistical analysis was performed with SPSS 26.0 (IBM Corp., Armonk, NY, USA).

## RESULTS

### Study sample

In total, 34 children participated in the program. Four children were excluded: 1 due to inclusion in an individual program and 3 due to health concerns. Finally, 30 patients met the inclusion criteria and were included in the study; 18 (60.0%) were girls. The mean age at the first visit to the MDT or an MDT member was 1.5 (1.8 SD) years. The mean age at the beginning of the program was 2.6 (1.9 SD) years. Before entering the program, the children had been treated by the MDT for a mean of 1.07 (0.9 SD) years.

Baseline characteristics are listed in Table 1. Several patients (n = 11, 36.0%) were prematurely born infants with multiple complications of prematurity. The majority (n = 21, 70.0%) had a psychomotor delay.

### Weaning from enteral nutrition

**Figure 1:**
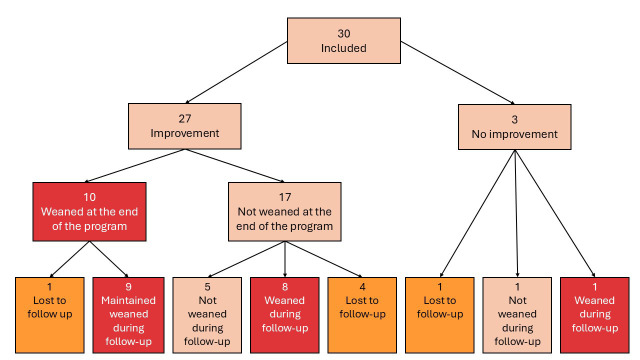
Study flowchart. Improvement is defined as an increase in oral intake.

Patient outcomes are presented in Figure 1. At the end of the program, 27 (90.0%) children improved their oral intake. Ten (33.3%) were completely weaned off EN, 17 (56.7%) improved but were not yet weaned at discharge, while 3 (10.0%) showed no improvement. During follow-up, an additional 8/17 children from this group were successfully weaned, while 5 remained on EN and 4 were lost to follow-up. Among the 3 children who showed no improvement during the program, one was weaned during follow-up, one remained dependent on EN, and one was lost to follow-up.

The mean duration of follow-up was 40.5 (25.7 SD) months. All patients who still required tube feeding at the end of follow-up had PEG placed instead of an NGT. None of the patients who were weaned off EN required reintroduction of EN during follow-up.

### Oral intake and anthropometry

A significantly higher proportion of patients increased their oral intake after the program ended and during follow-up compared with the beginning of the program. Furthermore, the enteral caloric intake significantly decreased after the end of the program (Table 2).

**Table 2:** Difference in the outcomes between four time points of the study*

	Program start (n = 30)	Program end (n = 30)	3-6 months of follow-up (n = 26)	Last visit (n = 24)	P
Any oral intake, n (%)	17 (56.7)	26 (86.7)	23 (88.5)	21 (87.5)	0.029
Weight for age Z score, mean (SD)	−1.3 (1.7)	−1.9 (2.3)	−1.8 (2.2)	−1.9 (1.8)	0.574
Height for age Z score, mean (SD)	−0.8 (2.2)	−1.0 (0.8)	−0.6 (1.5)	−1.1 (2.2)	0.836
Percentage of calories provided enterally, mean (SD)	90.1 (21.4)	38.7 (38.6)	31.6 (38.8)	21.4 (38.7)	<0.001*

*SD – standard deviation.

When we compared the patients who were weaned off EN after the program ended to those who were not weaned, only enteral caloric intake (as expected) at the end was significantly lower in the weaned group (Table 3).

**Table 3:** Differences between groups that were weaned and not weaned off enteral nutrition (EN) at the end of the two-week program

	Not weaned off EN (n = 20)	Weaned off EN (n = 10)	P
Age at beginning of the program, years (median, SD)	2.3 (1.3)	3.4 (2.6)	0.286
Sex, female (n, %)	11 (55.0)	7 (70.0)	0.247
Duration of NGT /PEG dependency, months (mean, SD)	27.0 (14.0)	36.9 (29.0)	0.257
Children with psychomotor delay, n (%)	15 (75.0)	6 (60.0)	0.105
Children with no oral intake, n (%)	12 (60.0)	5 (50.0)	0.118
Weight for age Z score at the beginning of the program (mean, SD)	−1.6 (1.8)	−0.8 (1.8)	0.267
Height for age Z score at the beginning of the program (mean, SD)	−1.4 (2.4)	−0.2 (1.8)	0.243
Percentage of calories given enterally at the beginning of the program (mean, SD)	92.0 (19.0)	84.0 (28.0)	0.488
Weight for age Z score at the end of the program (mean, SD)	−2.4 (2.6)	−1.4 (2.0)	0.320
Height for age Z score at the end of the program (mean, SD)	−1.0(0.9)	−0.8 (0.5)	0.802
Percentage of calories provided enterally at the end of the program (mean, SD)	52.0 (37.0)	0	0.001

*Abbreviations: NGT – nasogastric tube; PEG – percutaneous endoscopic gastrostomy; SD – standard deviation.

Our cohort experienced a non-significant reduction of 3.2% of initial body weight (mean −0.6 BW-for-age SD) at the end of the intervention. Anthropometric parameters (BW- and BH-for-age Z scores) remained constant after the end of the program (Table 2), without significant differences between the weaned and non-weaned patients (Table 3).

### Predictors of weaning success

Univariate logistic regression failed to demonstrate any of the evaluated factors (sex, age at inclusion, oral intake, psychomotor delay, mode of EN, and percentage of EN in total calorie requirements) as significant for a successful weaning off EN at the end of the program or at the end of follow-up.

## DISCUSSION

In our study, 10 out of 30 (33.3%) of the participants were fully weaned off EN at the end of the program, a number that increased to 18/24 (75.0%) at the end of follow-up. None of the weaned patients required reintroduction of EN during follow-up.

These results are in line with similar studies. A systematic review and meta-analysis by Sharp et al (8), including 11 studies with a total of 593 patients, showed an overall effect size of 71% for the percentage of patients successfully weaned off tube feeding at discharge, which increased to 80% at follow-up. Six studies (12-17) used hunger-induction as a mode of treatment, with all children being treated as inpatients. The programs lasted from a mean of 11.4 days up to three weeks, and in some studies, hospitalization was prolonged until the child was successfully weaned. All studies used a multidisciplinary treatment approach, although the exact approach varied. Compared with our study, tube-weaning in these studies was begun more aggressively, starting from 50% to 75% reduction in EN intake at the beginning of the program, further decreasing during hospitalization. These studies reported a 44% to 90% treatment success at the end of the program, and 63% to 90% at follow-up. Other studies using appetite manipulation, not included in the systematic review, showed short-term success rates varying from 51% to 100% (4,7,18-22) and long-term success rates varying from 74 to 92% (7,19-21). Two studies (18,22) used an outpatient hunger-induction tube-weaning approach, showing a success rate of 89.7% for the home-based approach (18) and 90.5% for the net coaching group (22). A lower rate of successfully weaned patients at the end of the program in our study can be explained by the day-hospital approach, together with a gradual tube feeding reduction of up to 50% before the start of the program and the less aggressive tube-weaning program lasting two weeks. Furthermore, our program included only patients who had already been treated by the MDT for a mean of 1.07 years and were resistant to previous standard interventions (individual behavioral and multidisciplinary treatment). Nevertheless, at follow-up, our program proved to be as successful as other described programs. However, it is not possible to determine from these results whether the outcomes can be attributed solely to the program itself or to other factors, such as the passage of time.

Tube-weaning interventions and programs differ considerably and have different success rates. For example, integrated behavioral interventions without hunger-induction showed a lower weaning rate at discharge (43%-90%) (9,13-15) and follow-up (64%-83%) (8) than hunger stimulation programs, which yielded significantly higher weaning success (70%) post-intervention (2).  Interventions in outpatient settings that incorporated multiple treatment factors (eg, multidisciplinary + behavioral + hunger provocation) demonstrated the greatest success (2). Interestingly, children in the inpatient/day-treatment setting increased the caloric intake by 82.6%, compared with only 70.5% in the outpatient setting (2). Unfortunately, no consensus on the best tube-weaning approach currently exists.

Weight loss during a tube-weaning intervention is expected and acceptable when amounting to 10% body weight (12,14-16). The short-term weight loss in studies that used appetite manipulation was 3.5%-9.3% (12,16-18). In our cohort, a non-significant reduction of 3.2% of initial body weight at the end of the program was detected. Greater weight loss in our cohort may have been prevented by medical monitoring, a multidisciplinary approach, and postponing further reduction of EN for children who were not clinically ready. Although weaning methods based on a more aggressive hunger stimulation and rapid weaning yield a higher percentage of weaned patients at discharge, weight loss is very common and, in some cases, severe (8). Moreover, some weaned patients require tube reintroduction due to the persistence of feeding disorders and inadequate weight gain. On the other hand, studies entailing a behavioral intervention require a longer duration and yield a lower weaning success rate at the end of the program, but cause less deterioration in anthropometric parameters (9,23).

In our cohort, none of the evaluated factors (sex, age at inclusion, peroral intake, psychomotor delay, mode of EN, and percentage of EN in the total calories at the start of the program) were significant for a successful weaning at the end of the program and follow-up. Trabi et al (17) showed that patients with PEG and children with more severe illnesses required a longer duration of weaning. Moreover, the chance of weaning increased if the tube was removed earlier in the treatment, while a higher BMI at admission prolonged the time needed for weaning. Sex and the degree of functional-emotional developmental delay did not influence weaning time (17). Nevertheless, insufficient data exist on the individual patient predisposing factors that could predict tube-weaning success.

The limitations of our study include the retrospective design, small sample size, and a heterogeneous cohort. The main strength of our study is the day-hospital approach, which has been reported only seldom. Day hospital care reduces the costs of the treatment and encourages caretakers’ involvement in the treatment from the start of the intervention.

In conclusion, a two-week day-hospital tube-weaning program effectively improved oral intake and the long-term tube-weaning success rate. The final goal of any intervention should be to provide a valuable and efficient tube-weaning model applicable in most centers. Our program encourages active participation of parents in every program activity. Tube weaning is accomplished by promoting behavioral changes along with hunger induction, allowing for persisting achievements.

Future research should prioritize randomized controlled trials to identify the most effective tube-weaning approaches for different patient populations. Additionally, exploring innovative models of care, including online and telehealth-based weaning programs, may offer promising alternatives to traditional in-person interventions in selected patients.
